# Adherence to 24-Hour Movement Guidelines among Spanish Adolescents: Differences between Boys and Girls

**DOI:** 10.3390/children8020095

**Published:** 2021-02-01

**Authors:** Miguel Angel Tapia-Serrano, Javier Sevil-Serrano, Pedro Antonio Sánchez-Miguel

**Affiliations:** 1 Department of Didactics of Musical, Plastic and Body Expression, Faculty of Teaching Training, University of Extremadura, Avenida Universidad, S/N, 10071 Cáceres, Spain; 2 Department of Didactics of Musical, Plastic, and Corporal Expression, Faculty of Health and Sport Sciences, University of Zaragoza, Plaza Universidad, 3, 22002 Huesca, Spain; jsevils@unizar.es

**Keywords:** physical activity, prevalence, 24-Hour Movement Guidelines, recommendations, screen time, sedentary, sex, sleep, youth

## Abstract

Background: The 24-Hour Movement Guidelines for adolescents recommend ≥60 min/day of moderate-to-vigorous physical activity (PA), ≤ 2 h/day of screen time, and 8–10 h/day of sleep. Since little information is available on the 24-Hour Movement Guidelines in Spanish adolescents, this study aims to estimate the proportion of Spanish adolescents meeting individual and combinations of these 24-Hour Movement Guidelines. Moreover, another aim of this study is to examine gender differences in compliance with 24-Hour Movement Guidelines. Methods: A final sample of 1465 Spanish adolescents (44.50% girls; 13.08 ± 0.86) participated in this cross-sectional study. The 24-Hour Movement Guidelines were measured during weekdays and the weekend days by self-reported questionnaires. Results: Although most adolescents met sleep duration guidelines (81.3%), only 38% and 15.8% met physical activity and screen time guidelines, respectively. Only 5.4% of these adolescents met all three 24-Hour Movement Guidelines, whereas 10.2% of this sample did not meet any of these guidelines. Although boys reported greater compliance with physical activity recommendations and girls with screen time recommendations, no significant gender differences were found in the compliance of all three 24-Hour Movement Guidelines. Conclusion: Given that 94.4% of Spanish adolescents did not meet 24-Hour Movement Guidelines, promoting all these three movement behaviours in both boys and girls is an urgent public health priority.

## 1. Introduction

It is well established that the adoption of a healthy lifestyle, characterized by high levels of physical activity, low levels of screen time, and optimal sleep duration, provides additional health benefits than the adoption of just one of these behaviours [1,2,3]. Although these health-related behaviours have usually been studied in isolation, compelling evidence shows that these movement behaviours interact across the whole day (24-hour period) [2,4,5,6]. Therefore, an increase in one of these movement behaviours (e.g., sleep duration) could be related to a decrease in other movement behaviours (e.g., physical activity), due to the finite amount of time in a single 24-hour period [7]. The 24-Hour Movement Guidelines for children and adolescents provide a new holistic approach to youth health promotion by integrating all 24-hour time-use continuum behaviours. Therefore, this integrative approach states that the whole day matters on health indicators [8,9]. 

These guidelines propose that adolescents should engage in at least 60 minutes per day of moderate-to-vigorous physical activity [8,10], should avoid spending more than 2 hours per day in leisure-time screen time (such as watching television, playing videogames, surfing the internet or social networks) [8,11], and should accumulate an optimal sleep duration per day (8–10 h/day) [5,8]. Adhering to all three 24-Hour Movement Guidelines has been related to more physical, social, and cognitive health benefits than meeting just one or none [3,9,12]. However, most existing studies have reported that less than 10% of adolescents met the 24-Hour Movement Guidelines [13,14,15,16,17,18,19,20,21,22,23,24,25,26,27,28,29,30,31,32]. These aforementioned studies have found inconsistent results in the proportion of adolescents who meet the 24-Hour Movement Guidelines, which have ranged from 0.3% to 2.0% in Asian [14,21,26] 3.1% in South Americans [25], 2.2% to 9.4% in North Americans (ranging from 2.2% to 5.2% in Canadian and from 5.0% to 9.4% in American) [4,15,16,18,22,23,24,28,29,30,31,32,33], and from 1.7% to 2.2% in European [17,19,27]. Most adolescents met sleep duration recommendations [13,14,15,17,18,19,20,21,22,25,28,29,30,31,32], whereas less than half of adolescents met physical activity [13,15,16,17,18,20,21,22,23,25,26,27,28,29,30,31] and screen time [14,19,32] recommendations in these mentioned studies.

Moreover, inconsistent results were also found in the proportion of adolescents who do not meet any of these three 24-Hour Movement Guidelines, which have ranged from 5.1% to 38.7% in Asian [14,21,26], 32.97% in South Americans [25], 9.1% to 42.0% in North Americans (ranging from 12.5% to 42.0% in Canadian and from 9.1% in American) [15,16,18,20,22,23,24,28,29,30,31,32,34], and from 8.7% to 36.4% in European [17,19,27]. These differences in 24-Hour Movement Guidelines across countries may be explained by social (e.g.; age and gender), cultural (e.g.; ethnicity), or economic (e.g.; socioeconomic status or family structure) factors [34]. Therefore, more research about the prevalence of these 24-Hour Movement Guidelines in different countries is required. 

To our knowledge, there is only one study that has analyzed the proportion of Spanish adolescents meeting the 24-Hour Movement Guidelines [19]. The study conducted by Sevil-Serrano et al. [19] showed that only 1.7% of participants met physical activity, screen time, and sleep duration recommendations, while 8.2% did not comply with any of these 24-Hour Movement Guidelines. Most of the adolescents of this study met sleep duration recommendations (88%), while only 21.4% and 1.7% of adolescents simultaneously met the physical activity and screen time recommendations, respectively. No gender differences in the meeting of 24-Hour Movement Guidelines were found in this study [19]. However, these findings cannot be generalized to the rest of the Spanish adolescents, because this study only included a small and non-representative sample of two high schools (*n* = 173) [19]. Given this limitation, more studies that examine the adherence to 24-Hour Movement Guidelines among Spanish adolescents are needed. In addition to the study mentioned above [19], only one study has examined gender differences in 24-Hour Movement Guidelines [26]. No gender differences in meeting physical activity, screen time, and sleep duration guidelines, separately or jointly, were found in this study [26]. 

Since little information is available on the 24-Hour Movement Guidelines in Spanish adolescents, this study has two aims: (1) to identify the proportion of Spanish adolescents meeting individual and combinations of these 24-Hour Movement Guidelines and; (2) to examine gender differences in compliance with 24-Hour Movement Guidelines.

## 2. Materials and Methods

### 2.1. Design and Participants

The present cross-sectional study was developed within a project aimed at promoting healthy behaviours among youth [35]. The baseline data were collected, before the COVID-19 pandemic, from March to June 2019 in Extremadura (Spain). In total, 2217 adolescents, from 22 high schools, initially participated for this study. Of the 2217 adolescents, 752 participants were excluded because physical activity (*n* = 122) and screen time (*n* = 630) were not reported, respectively. A finale sample of 1465 adolescents (652 girls; 13.05 ± 0.83 years, and 813 boys; 13.11 ± 0.89 years), aged between 11 to 16 years old (13.08 ± 0.86 years), participated in the study (student response rate was 66.1%).

This study was conducted in accordance with the Declaration of Helsinki and was approved by the Ethics Committee of the University of Extremadura (89/2016).

### 2.2. Measures

#### 2.2.1. 24-Hour Movement Guidelines

##### Physical Activity

Physical activity was measured using the Spanish version of the Physical Activity Questionnaire for Adolescents (PAQ-A; [36]). The instrument comprises nine items that relate to the type and frequency of participation in physical activities over the last 7 days. Participants self-reported the frequency of participation of a list of activities or moments such as physical education, school break, lunchtime, after school, evenings, and weekends. Each answer is scored on a 5-point scale ranging from 1 to 5. To compute the physical activity index score, the average value of all responses (higher scores indicate higher levels of physical activity) was calculated. The physical activity recommendations were categorized based on a cut-off score of 2.75 points to discriminate between "active" and "non-active" adolescents [37]. 

##### Screen Time 

Screen time was obtained using an adapted version of the Youth Leisure-Time Sedentary Behaviour Questionnaire (YLSBQ; [38]), validated in Spanish children and adolescents. The students self-reported their daily average time spent on four screen behaviours (i.e.: TV, videogames, computer, and mobile phone). The daily average time spent on each sedentary screen behaviour was calculated using a ratio of 5:2 (i.e., ((Daily screen time on weekend days × 5) + (Daily screen time on weekend days × 2)) ÷ 7). Total daily screen time was measured summing the different daily screen time behaviours.

##### Sleep Duration

Participants self-reported sleep duration for weekdays and weekend days. Daily sleep duration was assessed by weighting weekday and weekend day at a ratio of 5:2 (i.e., ((Daily sleep duration on weekday × 5) + (Daily sleep duration on weekend days × 2)) ÷ 7). 

The 24-Hour Movement Guidelines for adolescents recommend ≥60 min/day of moderate-to-vigorous physical activity, ≤2 h/day of screen time, and 8–10 h/day of sleep. Participants were classified into two groups for each movement behaviour: “meeting guidelines” and “not meeting guidelines” [39].

### 2.3. Procedure

The research team contacted the school principal and teachers to carry out this study. Parents were informed by letter about the nature and purpose of the study and written informed consent was required from both adolescents and their parents/legal guardians. The paper-and-pencil survey was administered by one member of the research team for approximately 30 minutes.

### 2.4. Statistical Analysis

All analyses were performed using SPSS version 23.0 (IBM, Armonk, NY, USA), and the level of significance was set at *p* < 0.05. Descriptive statistics were used to examine the daily average time spent in physical activity, screen time, and sleep duration, as well as the proportion of participants meeting individual 24-Hour Movement Guidelines (i.e.,: not meeting recommendations, meeting PA only, meeting ST only, and meeting SD only) and in combination (i.e., meeting PA + ST only, meeting PA + SD only, meeting ST + SD only, and meeting all three recommendations). Gender differences in 24-Hour Movement Guidelines were assessed using t Student’s for continuous variables and chi-square test for categorical variables. Given a significant interaction between physical activity, screen time, and sleep duration in relation to gender were found (all, *p* > 0.05), analyses were performed in both boys and girls.

## 3. Results

Descriptive characteristics of the participants and the prevalence of the three 24-Hour Movement Behaviours are presented in Table 1. The physical activity index score was found to be close to the mean value of the scale (2.53 of 5). Average screen time and sleep duration were 4.5 h/day and 8.65 h/day, respectively. Although boys reported higher physical activity levels than girls, no differences in screen time and sleep duration were found.

**Figure 1:**
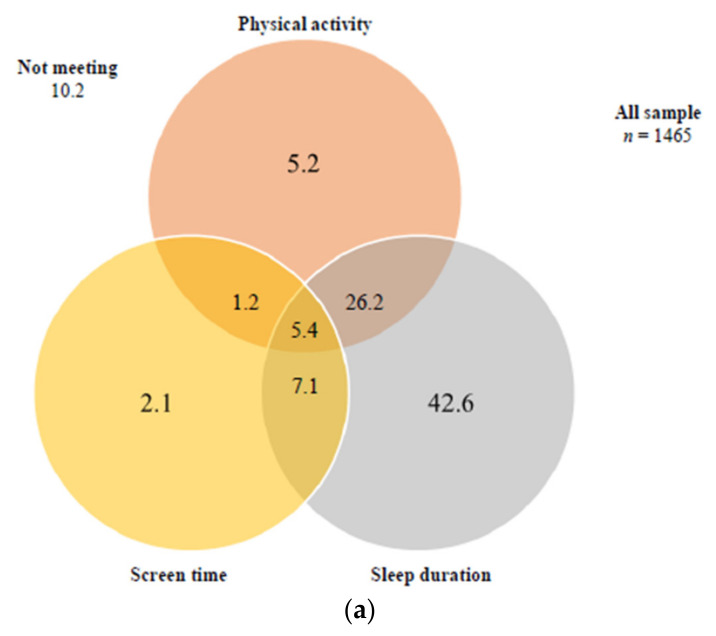

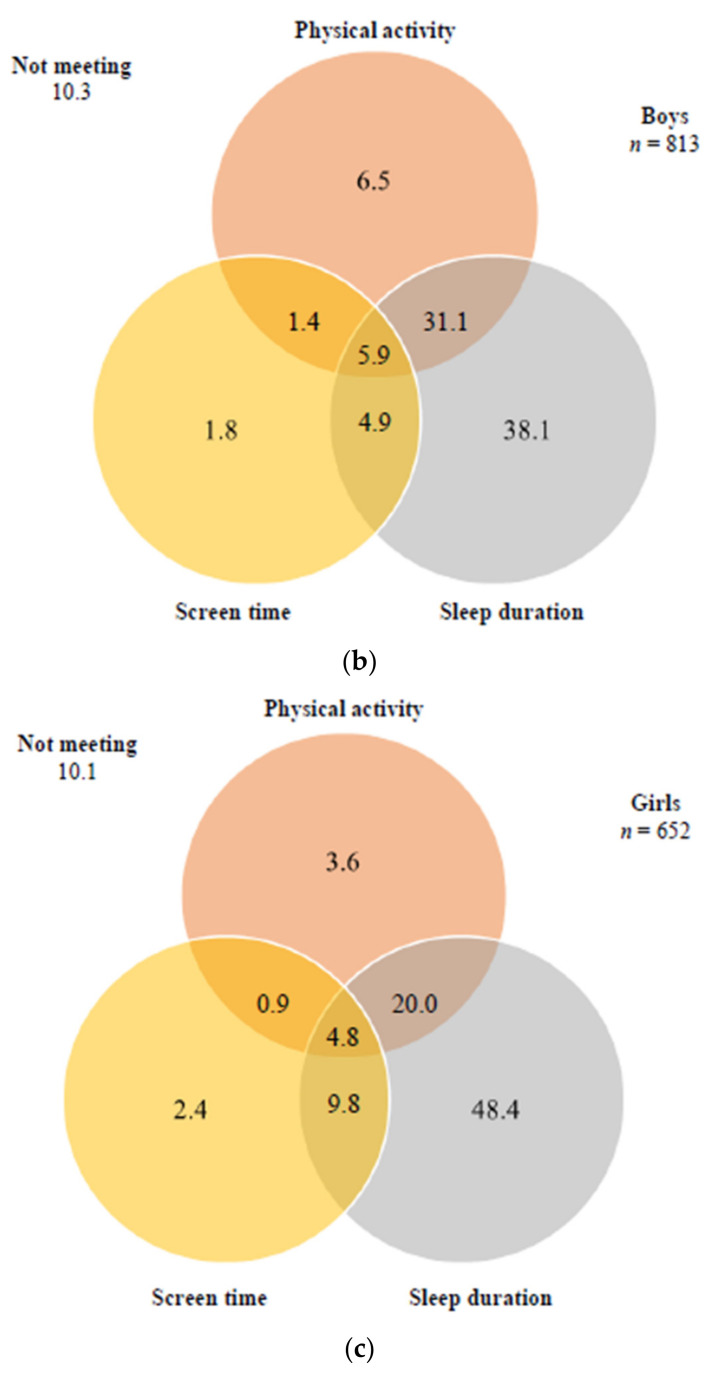
Venn diagram showing the proportion (%) of participants meeting 24-Hour Movement Guidelines separately and in all possible combinations. (**a**) Venn diagram showing the proportion (%) of participants meeting no guidelines; physical activity, sedentary screen time and sleep duration guidelines; and combinations of these guidelines, in the full study sample (*n* = 1465). Legend: recommendations are: ≥60min/day of moderate-to-vigorous physical activity; no more than 2 h/day of screen time and 8–10 h/day of sleep duration. The sum of each circle is equivalent to the % meeting each individual recommendation (i.e., 38.0% for physical activity, 15.7% for sedentary screen time and 81.3% for sleep duration); (**b**) Venn diagram showing the proportion (%) of participants meeting no guidelines; physical activity, sedentary screen time and sleep duration guidelines; and combinations of these guidelines, in boys (*n* = 813). The sum of each circle is equivalent to the % meeting each individual guideline (i.e., 44.9% for physical activity, 14.0% for sedentary screen time and 80% for sleep duration); (**c**) Venn diagram showing the proportion (%) of participants meeting no guidelines; physical activity, sedentary screen time and sleep duration guidelines; and combinations of these guidelines, in girls (*n* = 652). The sum of each circle is equivalent to the % meeting each individual guideline (i.e., 29.3% for physical activity, 17.9% for sedentary screen time and 83.0% for sleep duration).

Figure 1 shows the proportion of adolescents meeting 24-Hour Movement Guidelines separately and in all possible combinations. Most of the adolescents (81.3%) met sleep duration guidelines, but only 38% and 15.8% met physical activity and screen time guidelines, respectively. Only 10.2% of the sample did not meet any of these guidelines, 49.9% met exclusively one guideline, 34.5% met exclusively two guidelines, and, finally, 5.4% of the sample met all three guidelines. Boys reported significantly greater compliance with physical activity recommendations (6.5% vs. 3.6%) and the combination of physical activity and sleep duration recommendations (31.1% vs. 20.0%) than girls. Girls reported significantly greater compliance with screen time recommendations (2.4% vs. 1.8%) and the combination of screen time and sleep duration recommendations (9.8% vs. 4.9%) than boys.

Moreover, as shown in Table 2, the proportion of girls who met exclusively one guideline was higher than boys. Otherwise, the number of boys who met exclusively two guidelines were greater than girls.

## 4. Discussion

The main findings of this study revealed that a low proportion of Spanish adolescents met combination of physical activity, screen time, and sleep duration guidelines. Moreover, boys reported a higher percentage of physical activity recommendations and the combination of physical activity and sleep duration recommendations than girls. Otherwise, the number of girls who met screen time recommendations and the combination of screen time and sleep duration recommendations were greater than boys.

Only 5.4% of adolescents (5.9% boys and 4.8% girls) met the three 24-Hour Movement Guidelines, while 10.2% of adolescents (10.3% of boys and 10.1% of girls) did not meet any of these three recommendations. The adherence to 24-Hour Movement Guidelines found in our study is higher than those found in the studies conducted with European [17,19,27] and Asian adolescents [14,21,26] and lower than several studies conducted in the United States (5.0%–9.4%) [16,22,23]. However, our results are similar to the results found in Canadian adolescents [15,18,28,29,30,31,32,33]. These results underline the importance of addressing these movement behaviours from an integrative and holistic approach to increase physical activity and sleep duration and decrease screen time [8]. Regarding gender, no significant differences were found in meeting all three 24-Hour Movement Guidelines in both and girls. In addition, while boys reported a higher prevalence of combined physical activity and sleep duration recommendations, girls revealed greater compliance with the combination of screen time and sleep duration recommendations. 

Less than half of the adolescents met physical activity recommendations (38.0%). Regarding gender, boys (44.9%) reported higher adherence to physical activity guidelines than girls (29.3%). According to our results, a previous study conducted with a 1.6 million population revealed that 77.6% of adolescents’ boys and 84.7 of adolescents’ girls did not meet physical activity guidelines [40]. This study showed that the gender differences for physical activity recommendation are getting bigger in the last fifteen years, especially in some high-income countries, such as Ireland and the United States. Therefore, promoting physical activity, particularly among girls, is one of the biggest challenging today. Given that global trends in meeting physical activity recommendations are decreasing, new policies and action plans are needed to promote and increase physical activity in adolescents, particularly in girls. There are examples such as This Girl Can campaign developed in the United Kingdom, whose aim was to develop social marketing campaigns combined with community-based interventions to increase physical activity levels in girls [41].

Screen time guidelines were only achieved by 15.8% of adolescents. The proportion of students met screen time guidelines varies substantially from one study to another [42]. Specifically, a range between 25.7% and 40.1% have been found in European adolescents ranging in age from 14 to 18 years [17,27]. However, our results are slightly better than those found in a recent study conducted in Spanish adolescents, which reported that only 1.7% met the screen time guidelines [19]. Differences in compliance with the screen recommendations between studies could be explained by the type of questionnaire used to measure screen time. While some studies have only assessed television viewing time, other studies have assessed a wide range of screen-based behaviours [42]. Previous research has revealed an increased time spent on screen-based sedentary behaviours such as television, computers, videogames, and, especially, mobile phones in adolescents from developed countries [42], which may explain why many adolescents do not meet screen time guidelines. Given the breakthrough of new technologies in developed countries, practitioners, and policymakers should reconsider new screen time guidelines for each screen-based sedentary behaviour [19]. Moreover, in this investigation, girls (17.9%) were more likely to meet screen time guidelines than boys (14.0%). Our results are consistent with those found in the study by Guthold et al. [40] who reported higher compliance with screen time recommendations in girls. Differences have also been found between boys and girls regarding screen media use [7,43]. Generally, boys tend to spend more time on video games and computers [7], while girls spend more time talking on the phone, listening to music and using social networks [43].

Most adolescents met sleep time recommendations (81.3%). Unlike physical activity and screen time recommendations, no gender differences were found for sleep duration guidelines. Other studies have shown that between 40.1% and 25.7% of European adolescents meet sleep duration recommendations [17,27]. However, a recent study conducted in Spanish adolescents also found that 89.0% of adolescents met sleep duration guidelines [19]. Although sleep duration was adequate for most adolescents, almost 20.0% of adolescents reported low sleep duration, negatively affecting their health. Sleep duration is critical for the well-being of children and adolescents [44]. Previous studies have shown that an of lack sleep duration during adolescence may results in symptoms of mental health diagnoses, including depression and anxiety [45], poor health behaviours decisions, including overeating, skipping exercise, or misusing drugs such as caffeine, nicotine, or other stimulants [46]. 

### 4.1. Practical Implications

A few students comply with the recommendations for physical activity, screen time and sleep duration. Thus strategies and programs to promote adherence to the 24-Hour Movement Guidelines among Spanish adolescents are needed. Given the low compliance of adolescents with physical activity recommendations and, especially, screen time recommendations, special attention should be paid to these behaviours.

In this sense, school can be an ideal environment to promote adolescents´ health-related behaviours [47,48,49]. School-based lifestyle interventions can involve teachers, school staff, and families in developing and implementing programs to promote health-related behaviours in young people. For example, parental restrictions on the use of media, particularly one hour before bedtime, encouraging physical activity during school breaks [50], and promoting physical activity outside of school and active commuting [51] may be some strategies that could be used to reallocate sedentary time to active behaviours or optimal sleep duration. 

### 4.2. Limitations, Future Perspectives, and Strengths 

This study has several limitations that future research should address. Firstly, although validated and reliable questionnaires were used, self-reported measures to assess the 24-Hour Movement Guidelines could underestimate or overestimate the results. Secondly, only four of the twelve items of YLSBQ were included in the present study (i.e., television, videogames, computer, and mobile phone). It must be noted that these four screen-based behaviours are those with the highest prevalence among Spanish adolescents [52]. Finally, although a large sample of Spanish adolescents was used, the sample was not representative of the whole Spanish adolescents. Moreover, the response rate to the study was low because some adolescents did not complete the entire questionnaire. Future studies should use a larger representative sample of Spanish adolescents and measure the different movement behaviours through objective measures such as accelerometers. Another research field of research is to examine the temporal stability of these 24-Hour Movement Guidelines of Spanish adolescents using a longitudinal design. Finally, multiple health behaviour change interventions in Spanish adolescents’ boys and girls are required to improve these behaviours. Despite these limitations, this study has some strengths that should be acknowledged. This research is the first to examine, in a large sample of Spanish adolescents, the adherence to 24-Hour Movement Guidelines. Moreover, very few studies have examined gender differences in 24-Hour Movement Guidelines.

## 5. Conclusions

This study has revealed that only 5.4% of the Spanish adolescents met the three 24-Hour Movement Guidelines, whereas 10.2% did not meet any recommendations. Although boys reported greater compliance with PA recommendations and girls with screen time recommendations, no significant gender differences in the compliance of all three 24-Hour Movement Guidelines were found. Finally, futures policies and interventions should promote the 24-Hour Movement Guidelines behaviours among boys and girls, following an integrated and holistic approach.

## Figures and Tables

**Table 1:** Descriptive characteristics of the participants and prevalence of the three 24-hour Movement Behaviours among boys and girls.

Study Variables	Total	Boys	Girls	*p*
	M ± SD	M ± SD	M ± SD	
*n* (%)	1465 (100.00)	813 (55.50)	652 (44.50)	
Age groups *n* (%)				
1st graders (11–12 years)	362 (24.70)	198 (24.40)	164 (25.20)	0.239
2nd graders (13–14 years)	682 (46.60)	367 (45.10)	315 (48.30)	
3rd graders (15–16 years)	421 (28.70)	248 (30.50)	173 (26.50)	
Physical activity (1–5)	2.53 ± 0.60	2.63 ± 0.59	2.40 ± 0.58	<0.001
Screen time (h/day)	4.51 ± 2.26	4.62 ± 2.21	4.39 ± 2.33	0.054
Sleep duration (h/day)	8.65 ± 0.93	8.64 ± 0.98	8.66 ± 0.87	0.608

**Table 2:** Meeting of 24-Hour Movement Guidelines among adolescents’ boys and girls.

Meeting 24-Hour Movement Guidelines	Total	Boys	Girls	*p*
	*n* (%)	*n* (%)	*n* (%)	
Not meeting guidelines	150 (10.20)	84 (10.30)	66 (10.10)	0.483
Meeting exclusively 1 guideline	731 (49.90)	377 (46.40)	354 (54.30)	<0.010
PA	556 (38.00)	365 (44.90)	191 (29.30)	<0.001
ST	200 (15.80)	114 (14.00)	117 (17.90)	<0.050
SD	1191 (81.30)	650 (80.00)	541 (83.00)	0.079
Meeting exclusively 2 guidelines	505 (34.50)	304 (37.40)	201 (30.80)	<0.010
PA + ST	96 (6.60)	59 (7.30)	37 (5.70)	0.133
PA + SD	433 (31.60)	280 (37.00)	153 (24.80)	<0.001
ST + SD	178 (12.50)	87 (10.80)	91 (14.60)	<0.050
Meeting 3 guidelines				
PA + ST + SD	79 (5.40)	48 (5.90)	31 (4.80)	0.198

PA: physical activity; ST: screen time; SD: sleep duration.

## Data Availability

Authors will be happy to share data analyses to with those who request it.
